# An Integrated Review of Conventional and Emerging Diagnostic and Therapeutic Modalities to Reduce the Risk of Surgical Resections in Intestinal Tuberculosis

**DOI:** 10.3390/diagnostics16091332

**Published:** 2026-04-29

**Authors:** Khalid Alyahyawi

**Affiliations:** Department of Surgery, College of Medicine, Jazan University, Jazan 45142, Saudi Arabia; kyahyawi@jazanu.edu.sa

**Keywords:** intestinal tuberculosis, molecular diagnostics, endoscopy, GeneXpert, artificial intelligence, CRISPR, anti-tubercular therapy, minimally invasive surgery

## Abstract

Intestinal tuberculosis (ITB) is a complex form of extrapulmonary tuberculosis characterized by nonspecific gastrointestinal symptoms and substantial overlap with conditions such as Crohn’s disease and gastrointestinal malignancies. These similarities frequently lead to diagnostic uncertainty, delayed diagnosis, and inappropriate management. This systematic review summarizes current evidence on the clinical presentation, diagnostic approaches, and therapeutic strategies for ITB, with particular emphasis on emerging diagnostic technologies and their role in reducing surgical interventions. A systematic literature search was conducted using PubMed, Scopus, and Google Scholar following PRISMA guidelines to identify relevant studies published from 2000 to 2025. The review focused on clinical manifestations, imaging findings, endoscopic features, histopathological characteristics, molecular diagnostics, pharmacological therapy, and minimally invasive therapeutic interventions. Accurate diagnosis requires an integrated approach combining clinical assessment with imaging, endoscopic evaluation, and histopathological confirmation. Molecular techniques such as GeneXpert MTB/RIF and GeneXpert MTB/RIF Ultra and multiplex polymerase chain reaction assays improve diagnostic accuracy and shorten detection time. Emerging technologies including artificial intelligence-assisted radiologic interpretation and CRISPR-based stool sequencing platforms show promise for earlier detection. Standard anti-tubercular therapy remains the cornerstone of treatment, while minimally invasive endoscopic and surgical procedures are effective for managing complications such as strictures, obstruction, and perforation. Early and precise diagnosis of intestinal tuberculosis is essential to prevent complications and optimize patient outcomes. Integrating conventional diagnostic approaches with emerging molecular and artificial intelligence-based technologies may enhance diagnostic precision and support individualized treatment strategies. Further ITB-specific clinical studies are needed to validate novel diagnostic tools and refine therapeutic approaches for improved patient care.

## 1. Introduction

Intestinal tuberculosis (ITB) is an important form of extrapulmonary tuberculosis that affects the gastrointestinal tract. The disease most commonly involves the ileocecal region, although any segment of the small or large intestine may be affected [1,2]. Extrapulmonary tuberculosis accounts for approximately 15–20% of all tuberculosis cases worldwide, and intestinal involvement represents a substantial proportion of abdominal tuberculosis, particularly in high-burden regions [1]. The burden of ITB remains highest in Southeast Asia, the Middle East, sub-Saharan Africa, and parts of Latin America, where tuberculosis incidence exceeds 100 cases per 100,000 population. In these endemic areas, ITB accounts for approximately 1–3% of all tuberculosis cases and up to 10–15% of extrapulmonary tuberculosis cases [1,2].

In industrialized countries, the incidence of ITB is lower but has shown a gradual increase. This trend is largely associated with migration from endemic regions and the increasing prevalence of immunocompromised populations. Individuals with HIV infection, diabetes mellitus, or malnutrition or those receiving immunosuppressive therapy are particularly susceptible to intestinal tuberculosis [3]. Understanding the epidemiology, risk factors, and clinical manifestations of ITB is essential for early detection and appropriate management.

Clinically, ITB is often described as a “great mimic” because of its highly variable and nonspecific presentation. Patients are commonly present with chronic abdominal pain, weight loss, fever, altered bowel habits, gastrointestinal bleeding, or signs of intestinal obstruction. These symptoms overlap substantially with Crohn’s disease and gastrointestinal malignancies, creating considerable diagnostic challenges and frequently delaying accurate diagnosis [3,4]. Recognized risk factors include previous or concurrent pulmonary tuberculosis, HIV infection, malnutrition, diabetes mellitus, close contact with individuals with tuberculosis, and residence in endemic settings [2,3,5].

Diagnosis of ITB relies on the integration of clinical findings with radiologic imaging, endoscopic evaluation, microbiological testing, and histopathological examination of intestinal biopsy specimens. Conventional diagnostic approaches include contrast-enhanced computed tomography, endoscopic biopsy, acid-fast bacilli staining, and mycobacterial culture. Advances in diagnostic technologies, particularly molecular assays such as polymerase chain reaction and GeneXpert MTB/RIF, have improved diagnostic sensitivity and reduced time to detection [6,7]. If not diagnosed and treated promptly, intestinal tuberculosis may progress to severe complications including strictures, perforation, fistula formation, bowel obstruction, and gastrointestinal hemorrhage. These complications frequently require surgical intervention and are associated with increased morbidity and prolonged hospitalization [8].

Although standard four-drug anti-tubercular therapy remains the cornerstone of treatment, medical therapy alone may not always prevent inflammatory complications that ultimately require surgical management [8,9]. Significant uncertainty remains regarding the optimal integration of advanced diagnostic modalities with adjunctive therapeutic strategies aimed at reducing disease progression and surgical risk. Addressing this gap requires improved diagnostic sequencing involving imaging, endoscopy, and molecular testing, together with evaluation of adjunctive host-directed therapies capable of modulating inflammatory responses associated with disease progression [4]. Given these challenges, preventing progression to surgical disease remains a critical objective in the management of ITB. Multidisciplinary care that includes early diagnosis, optimized anti-tubercular therapy, minimally invasive interventions, and supportive management strategies offers the potential to reduce complications and improve clinical outcomes. This systematic review provides an evidence-based overview of intestinal tuberculosis with a focus on contemporary diagnostic strategies and evolving management approaches that aim to reduce the need for surgical intervention.

## 2. Materials and Methods

### 2.1. Literature Search Strategy

A systematic literature review was conducted in accordance with PRISMA (Preferred Reporting Items for Systematic Reviews and Meta-Analyses) guidelines to summarize current evidence on the diagnosis and management of intestinal tuberculosis (ITB). Relevant studies published between 2000 and 2025 were identified through searches of PubMed, PubMed Central, ScienceDirect, and Google Scholar. The search strategy incorporated combinations of keywords and Medical Subject Headings (MeSH) including “intestinal tuberculosis”, “abdominal tuberculosis”, “diagnosis”, “GeneXpert”, “PCR”, “endoscopic ultrasound”, “artificial intelligence imaging”, “cine-MRI”, “stricture management”, “endoscopic therapy”, “anti-tubercular therapy”, “host-directed therapy”, and “paradoxical reaction”. Boolean operators (AND/OR) were used to refine the search. A complete list of search keywords organized by thematic category is provided in Appendix A.2. 

### 2.2. Eligibility Criteria

Studies were included if they addressed diagnostic modalities, therapeutic strategies, minimally invasive or endoscopic interventions, or management approaches relevant to intestinal tuberculosis. Particular emphasis was placed on studies evaluating imaging modalities, endoscopic techniques, histopathology, molecular diagnostic tools, and pharmacological treatments that may contribute to reducing disease progression and the need for surgical intervention. Articles were excluded if they were non-English publications, animal studies, conference abstracts without full text, narrative commentaries lacking primary data, or reports not directly related to the diagnosis or management of intestinal tuberculosis.

### 2.3. Study Selection and Data Synthesis

Titles and abstracts were screened to identify potentially relevant studies. Full texts of eligible articles were subsequently reviewed. Relevant findings were extracted and synthesized narratively (see Appendix A.1 for full study characteristics) to provide an overview of current diagnostic approaches, emerging technologies, and therapeutic strategies related to intestinal tuberculosis. Emphasis was placed on evidence that contributes to improved diagnostic accuracy, earlier detection, and reduction in complications requiring surgical intervention. The study selection process was documented according to PRISMA guidelines, including identification, screening, eligibility, and inclusion phases. In-text citations were numbered sequentially throughout the manuscript, and the reference list was ordered accordingly.

## 3. Results

### 3.1. Search Results and Study Inclusion

**Figure 1:**
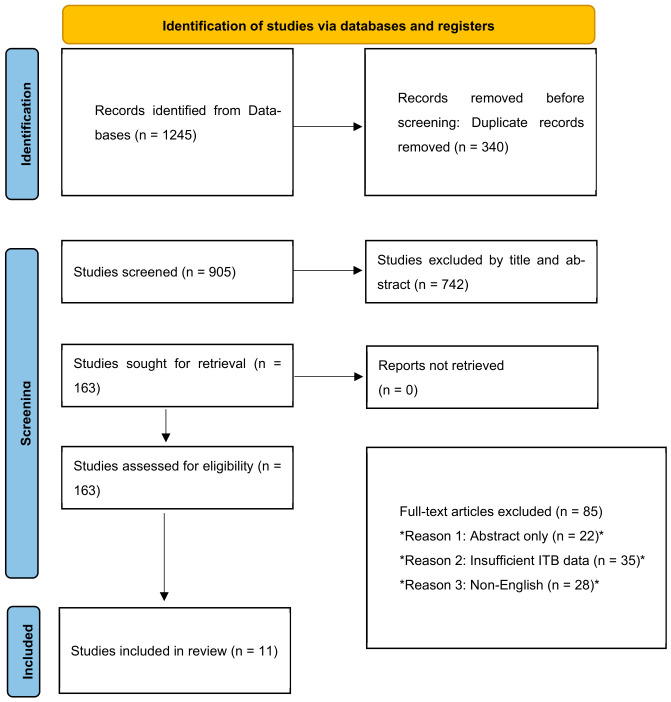
PRISMA flow diagram of study selection process.

The systematic search identified a total of 1245 records. After removal of 340 duplicates, 905 records were screened based on title and abstract. Of these, 742 records were excluded as they did not meet the inclusion criteria (e.g., non-human studies, pulmonary TB focus, case reports without diagnostic data). The full texts of 163 articles were assessed for eligibility. Subsequently, 85 articles were excluded for reasons including: abstract only (n = 22), insufficient data on intestinal TB outcomes (n = 35), and non-English language (n = 28). Ultimately, 78 studies met the inclusion criteria and were included in this systematic review. The selection process is summarized in Figure 1 (PRISMA 2020 Flow Diagram).

### 3.2. Study Characteristics

The 78 included studies comprised 23 retrospective cohort analyses, 18 prospective diagnostic accuracy trials, 15 cross-sectional studies, 12 systematic reviews/meta-analyses, and 10 clinical guideline documents. Publication dates ranged from 2000 to 2025, with a concentration of molecular diagnostic studies (n = 22) appearing after 2018. Geographically, 58% of studies originated from high-burden TB regions (Southeast Asia, Africa), ensuring applicability to endemic settings. A detailed summary of the characteristics, key findings, and limitations of all 78 included studies is provided in Appendix A.1.

### 3.3. Clinical Presentation and Risk Factors

Intestinal tuberculosis presents a wide spectrum of clinical manifestations that frequently overlap with other gastrointestinal disorders. The disease most commonly affects the ileocecal region, although involvement of other segments of the gastrointestinal tract may occur [10]. The clinical course is typically gradual, with symptoms developing over several weeks or months.

Patients are commonly present with chronic abdominal pain, weight loss, low-grade fever, anorexia, and changes in bowel habits. Diarrhea or constipation may occur, and some individuals experience alternating bowel patterns. In advanced disease, complications such as intestinal obstruction, gastrointestinal bleeding, or perforation may develop [8]. Because these symptoms are nonspecific, intestinal tuberculosis is frequently misdiagnosed as inflammatory bowel disease, particularly Crohn’s disease [11]. Several studies have reported that patients initially diagnosed with Crohn’s disease were later found to have intestinal tuberculosis after further diagnostic evaluation or response to anti-tubercular therapy [12]. Misdiagnosis remains a significant clinical challenge, particularly in regions where both diseases coexist. Systemic manifestations may accompany gastrointestinal symptoms, especially in patients with disseminated tuberculosis. These include fatigue, night sweats, and generalized malaise. Although these features may raise suspicion for tuberculosis, they are not specific to intestinal involvement [13].

Several risk factors increase the likelihood of developing intestinal tuberculosis. Previous or concurrent pulmonary tuberculosis is one of the most important clinical indicators. Additional risk factors include human immunodeficiency virus infection, diabetes mellitus, malnutrition, and close contact with individuals infected with tuberculosis [14]. Socioeconomic conditions and residence in endemic regions also contribute to increased exposure and disease risk. Given the nonspecific nature of clinical manifestations, recognition of epidemiological risk factors plays an important role in raising clinical suspicion. Early identification of high-risk patients facilitates timely diagnostic investigation and appropriate therapeutic management [15].

## 4. Diagnostic Modalities in Intestinal Tuberculosis

Accurate diagnosis of intestinal tuberculosis requires a multidisciplinary approach that integrates clinical assessment with radiologic imaging, endoscopic evaluation, histopathological analysis, and microbiological or molecular testing. Because the clinical manifestations overlap with several gastrointestinal disorders, particularly Crohn’s disease, a single diagnostic test is rarely sufficient. Instead, diagnosis typically relies on a combination of complementary modalities that together improve diagnostic accuracy and reduce delays in treatment initiation [16,17].

### 4.1. Radiologic Imaging

Radiologic imaging plays a central role in the initial evaluation of suspected intestinal tuberculosis. Abdominal ultrasound is often used as a preliminary imaging modality, especially in resource-limited settings, where it may reveal bowel wall thickening, ascites, or mesenteric lymphadenopathy [18,19]. However, the specificity of ultrasound findings is limited, and further imaging is often required. Computed tomography and CT enterography provide more detailed evaluation of intestinal involvement. Typical radiologic findings include circumferential bowel wall thickening, narrowing of the ileocecal region, necrotic lymph nodes, and inflammatory changes in the surrounding mesentery [20,21,22]. These imaging modalities are also valuable in identifying complications such as obstruction, fistula formation, and perforation.

### 4.2. Endoscopic Evaluation

Endoscopic examination is an essential diagnostic tool because it allows direct visualization of mucosal abnormalities and enables tissue sampling for histopathological and microbiological evaluation. Colonoscopy with ileoscopy is particularly useful because the ileocecal region represents the most commonly involved site in intestinal tuberculosis. Endoscopic findings may include transverse ulcers, nodularity, mucosal edema, strictures, and pseudopolyps. These findings are not specific and may resemble inflammatory bowel disease, particularly Crohn’s disease [23]. Therefore, obtaining multiple biopsies from suspicious lesions is critical to improve diagnostic yield. Endoscopic ultrasound has also been investigated as an adjunct diagnostic modality. This technique enables detailed visualization of bowel wall layers and adjacent lymph nodes and may facilitate fine-needle aspiration for cytological analysis in selected cases [24,25].

### 4.3. Histopathology and Microbiological Diagnosis

Histopathological examination of intestinal biopsy specimens remains a cornerstone of diagnosis. The presence of granulomatous inflammation with caseous necrosis strongly supports the diagnosis of intestinal tuberculosis, although granulomas may occasionally be absent or nonspecific [7]. Acid-fast bacilli staining may demonstrate mycobacteria within tissue specimens, but its sensitivity is relatively low because intestinal tuberculosis often represents a paucibacillary disease. Mycobacterial culture remains the gold standard for confirming Mycobacterium tuberculosis infection, although culture results may require several weeks to become available [26].

### 4.4. Molecular Diagnostic Techniques

Molecular diagnostic assays have significantly improved detection of Mycobacterium tuberculosis in intestinal tissue samples. Polymerase chain reaction-based assays enable rapid identification of mycobacterial DNA and may increase diagnostic sensitivity when conventional staining and culture methods are negative [27]. The GeneXpert MTB/RIF assay represents an important advance in tuberculosis diagnostics because it simultaneously detects Mycobacterium tuberculosis and rifampicin resistance within a short time frame. This rapid molecular test facilitates early diagnosis and initiation of appropriate therapy [6,7,28]. The GeneXpert MTB/RIF Ultra assay, which is recommended by the World Health Organization for extrapulmonary tuberculosis, offers improved sensitivity with a lower limit of detection (LOD) of 15.6 CFU/mL compared to 112.6 CFU/mL for the standard Xpert MTB/RIF assay, making it particularly valuable for paucibacillary specimens commonly encountered in intestinal tuberculosis [29,30].

### 4.5. Sample Collection and Processing for Diagnostic Testing

Appropriate sample acquisition is essential for accurate diagnosis of intestinal tuberculosis. Tissue samples are typically obtained during endoscopic evaluation, with multiple biopsies recommended from the ulcer base, ulcer edge, and surrounding mucosa to maximize diagnostic yield. At least 6–8 biopsy specimens should be collected from suspicious lesions when ITB is suspected [31]. Additionally, ascitic fluid, when present, can be obtained via paracentesis for biochemical, cytological, and molecular analysis. Lymph node sampling may be performed through endoscopic ultrasound-guided fine-needle aspiration or biopsy when mesenteric or retroperitoneal lymphadenopathy is identified. In cases where endoscopic access is limited, image-guided percutaneous biopsy of accessible lesions may be considered. All collected specimens should be submitted for histopathological examination, acid-fast bacilli staining, mycobacterial culture, and molecular testing. Notably, diagnostic testing is typically performed simultaneously rather than sequentially once samples are obtained, allowing for integrated interpretation of results from multiple modalities to establish a composite diagnosis [32].

### 4.6. Diagnostic Test Performance

The sensitivity and specificity of various diagnostic modalities for intestinal tuberculosis vary considerably depending on the test type and specimen quality. A summary of the diagnostic performance characteristics of commonly used tests is presented in Table 1. Serum biomarkers, including adenosine deaminase (ADA), have demonstrated utility particularly in peritoneal tuberculosis, with elevated ADA levels in ascitic fluid serving as a valuable diagnostic marker [33]. Serum ADA levels may also be elevated in intestinal tuberculosis, though with lower sensitivity compared to peritoneal involvement.

### 4.7. Emerging Diagnostic Technologies

**Figure 2:**
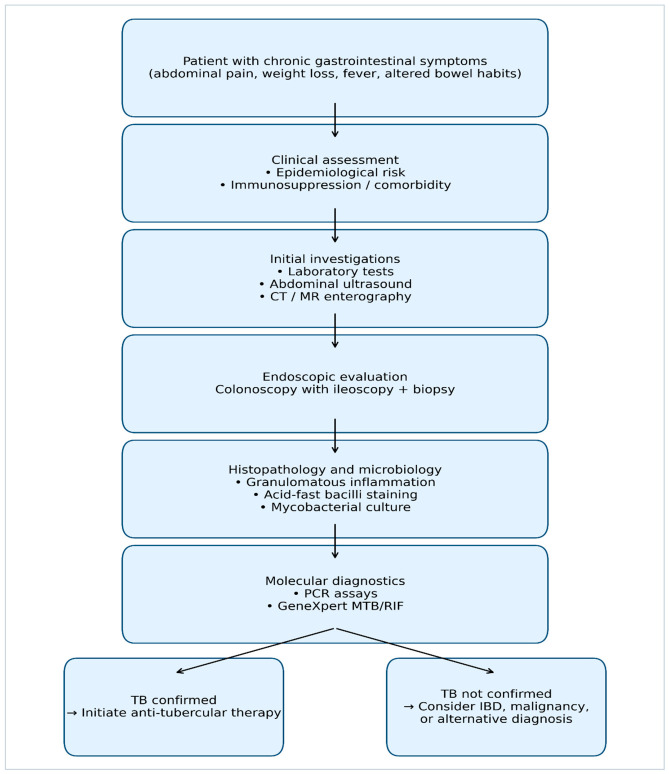
Diagnostic Algorithm for Intestinal Tuberculosis. Clinical diagnosis may be established in the absence of microbiological confirmation based on composite assessment including epidemiology, clinical features, imaging findings, endoscopic appearance, histopathology, and response to anti-tubercular therapy. Treatment initiation should be considered when clinical suspicion is high, even with negative microbiological results.

Recent advances in diagnostic technologies have introduced new approaches for improving diagnostic accuracy. Multiplex molecular assays and advanced PCR techniques may enhance pathogen detection and help differentiate intestinal tuberculosis from other inflammatory diseases [34]. Emerging platforms such as CRISPR-based targeted sequencing combined with next-generation sequencing have also been investigated as potential diagnostic tools capable of detecting mycobacterial DNA and identifying drug resistance mutations [35,36]. Artificial intelligence and machine learning techniques are increasingly being applied to medical imaging and histopathology. Recent studies have demonstrated promising results in differentiating intestinal tuberculosis from Crohn’s disease using deep learning analysis of endoscopic images and whole-slide histopathology [37,38,39]. A diagnostic algorithm for intestinal tuberculosis, emphasizing the composite nature of diagnosis and the option for clinical diagnosis with empiric treatment initiation in the absence of microbiological confirmation, is illustrated in Figure 2.

**Table 1:** Sensitivity and Specificity of Diagnostic Tests for Intestinal Tuberculosis.

Diagnostic Test	Sensitivity (%)	Specificity (%)	References
Acid-fast bacilli smear (tissue)	5–35	95–100	[40,41,42]
Mycobacterial culture (tissue)	15–45	98–100	[40,41,43]
Histopathology (caseating granulomas)	35–65	85–95	[40,42,44]
PCR (tissue)	55–80	85–95	[27,41,45]
GeneXpert MTB/RIF (tissue)	45–75	95–100	[6,7,46]
GeneXpert MTB/RIF Ultra (tissue)	60–85	95–100	[29,47]
Serum ADA	55–75	70–90	[33,48]
Ascitic fluid ADA	85–95	85–95	[33,49]
Fecal calprotectin	70–85 *	40–60 *	[50,51,52]

* Sensitivity and specificity for detecting intestinal inflammation; not specific for distinguishing ITB from other inflammatory conditions. ADA: adenosine deaminase; PCR: polymerase chain reaction.

## 5. Differential Diagnosis Between Intestinal Tuberculosis and Crohn’s Disease

**Figure 3:**
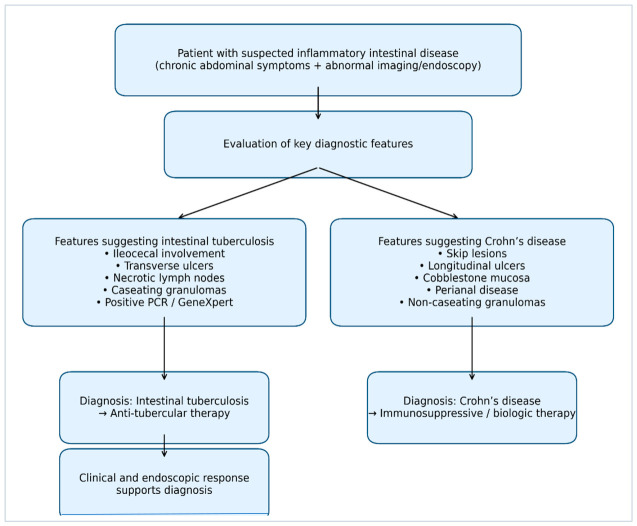
Diagnostic Differentiation Between Intestinal TB and Crohn’s Disease.

Differentiating intestinal tuberculosis from Crohn’s disease remains one of the most significant diagnostic challenges in gastroenterology. Both conditions share overlapping clinical, endoscopic, radiologic, and histopathological features, which often leads to misdiagnosis and delays in appropriate treatment. This distinction is particularly important because the therapeutic strategies for these diseases differ substantially; inappropriate immunosuppressive therapy for Crohn’s disease in patients with undiagnosed tuberculosis may lead to severe complications and disease progression [11,12]. Key clinical, endoscopic, radiologic, histopathological, and laboratory features that help differentiate intestinal tuberculosis from Crohn’s disease are summarized in Table 2 and Figure 3.

Clinically, both diseases may be presented with chronic abdominal pain, weight loss, fever, diarrhea, and intestinal obstruction. However, certain clinical characteristics may provide clues for differentiation. Patients with intestinal tuberculosis more frequently present with systemic symptoms such as fever and night sweats, whereas Crohn’s disease is more commonly associated with chronic diarrhea and perianal disease [2].

Radiologic imaging also plays a valuable role in distinguishing these conditions. In intestinal tuberculosis, imaging studies often demonstrate involvement of the ileocecal region, necrotic lymphadenopathy, and concentric bowel wall thickening. In contrast, Crohn’s disease typically presents with skip lesions, asymmetric bowel wall thickening, and creeping mesenteric fat [20,21,22].

Endoscopic findings may also assist in the diagnostic process. Intestinal tuberculosis frequently presents with transverse ulcers, nodular mucosa, and short-segment strictures, whereas Crohn’s disease more commonly demonstrates longitudinal ulcers, cobblestone appearance, and aphthous lesions [16,23].

Histopathological examination remains one of the most important diagnostic tools. Caseating granulomas strongly support the diagnosis of intestinal tuberculosis, whereas non-caseating granulomas are more typical of Crohn’s disease. However, granulomas may be absent in both conditions, and biopsy samples may not always provide definitive differentiation [23].

Because no single diagnostic test is completely reliable, clinicians often rely on a combination of clinical features, imaging findings, endoscopic appearance, histopathology, and microbiological testing to establish the diagnosis. In uncertain cases, a therapeutic trial of anti-tubercular therapy with close clinical and endoscopic follow-up may assist in distinguishing intestinal tuberculosis from Crohn’s disease [16]. Because intestinal tuberculosis and Crohn’s disease share several clinical and endoscopic features, differentiation often requires integration of radiologic, histopathological, and microbiological findings.

## 6. Therapeutic Strategies for Intestinal Tuberculosis

Management of intestinal tuberculosis primarily relies on pharmacological therapy with anti-tubercular drugs. Early initiation of treatment is essential to prevent disease progression and reduce the risk of complications such as intestinal obstruction, strictures, fistula formation, or perforation. Treatment strategies typically combine medical therapy with supportive care and, in selected cases, minimally invasive or surgical interventions [53].

### 6.1. Anti-Tubercular Therapy

Standard anti-tubercular therapy (ATT) remains the cornerstone of intestinal tuberculosis management. Clinical improvement often occurs within several weeks after initiation of therapy, with reduction in abdominal symptoms and gradual healing of intestinal lesions. In many patients, therapeutic response itself may support the diagnosis when microbiological confirmation is not obtained [54]. The recommended regimen generally consists of a four-drug combination including isoniazid, rifampicin, pyrazinamide, and ethambutol during the intensive phase, followed by a continuation phase with isoniazid and rifampicin [55]. Treatment duration typically ranges from six to nine months, although some studies suggest that a six-month regimen may be sufficient in most patients when adequate clinical response is achieved [55].

### 6.2. Management of Drug-Resistant Tuberculosis

Drug-resistant tuberculosis represents an emerging challenge in the management of extrapulmonary tuberculosis. Multidrug-resistant tuberculosis (MDR-TB), defined as resistance to at least isoniazid and rifampicin, requires alternative treatment regimens that include second-line agents such as fluoroquinolones, bedaquiline, or delamanid [56]. The World Health Organization currently recommends shorter all-oral bedaquiline-containing regimens for MDR-TB, with treatment duration ranging from 9 to 11 months in eligible patients. For extensively drug-resistant tuberculosis (XDR-TB) or complicated MDR-TB cases, individualized longer regimens lasting 18–20 months may be required, incorporating newer agents such as pretomanid and linezolid in combination with bedaquiline [57,58]. Recent studies have demonstrated promising outcomes with bedaquiline-containing regimens in patients with drug-resistant tuberculosis, although data specific to intestinal involvement remain limited.

### 6.3. Adjunctive and Host-Directed Therapies

Adjunctive therapies aimed at modulating host immune responses have been investigated to improve treatment outcomes and reduce inflammatory complications. Immunomodulatory approaches, including the use of agents such as Mycobacterium indicus pranii, have been explored as potential adjuncts to conventional anti-tubercular therapy [59,60]. These therapies aim to enhance immune-mediated clearance of mycobacteria while limiting tissue damage associated with excessive inflammatory responses. In addition, host-directed therapeutic strategies targeting inflammatory pathways are currently under investigation and may provide future treatment options for tuberculosis [61].

### 6.4. Paradoxical Reactions and Immune Reconstitution

Some patients undergoing anti-tubercular therapy may experience paradoxical reactions, characterized by clinical or radiologic worsening despite appropriate treatment. These reactions are believed to result from immune reconstitution or exaggerated inflammatory responses during therapy. Management of paradoxical reactions may require anti-inflammatory therapy, including corticosteroids in selected cases, particularly when severe inflammatory complications occur [62]. Such reactions may present as new or enlarging lesions, intestinal perforation, or worsening obstruction, as described in several clinical reports [63,64].

### 6.5. Treatment Monitoring and Follow-Up

Monitoring of treatment response in intestinal tuberculosis involves a combination of clinical assessment, laboratory parameters, endoscopic evaluation, and imaging studies. Clinical follow-up should be conducted at regular intervals, typically monthly during the intensive phase and every 2–3 months during the continuation phase. Key clinical parameters to monitor include resolution of abdominal pain, weight gain, normalization of bowel habits, and absence of fever [65]. Endoscopic reassessment is generally recommended after completion of 2–3 months of therapy to document mucosal healing and may be repeated at treatment completion if initial findings were severe or complications were present. Radiologic monitoring with CT enterography or ultrasound may be performed at 3–6-month intervals to assess regression of bowel wall thickening, lymphadenopathy, and resolution of ascites [20]. For patients with drug-resistant tuberculosis, more intensive monitoring is required, including monthly sputum examination for pulmonary co-infection, regular liver and renal function tests, electrocardiographic monitoring for patients receiving bedaquiline or fluoroquinolones, and periodic audiological assessment for those on aminoglycosides [57]. Treatment failure or relapses should be suspected if clinical symptoms persist or recur after initial improvement, if endoscopic lesions fail to heal, or if new complications develop during therapy.

## 7. Minimally Invasive and Surgical Management

Although most patients with intestinal tuberculosis respond well to anti-tubercular therapy, a subset of patients develop complications that require interventional or surgical management. These complications may include intestinal obstruction, fibrotic strictures, perforation, fistula formation, or abscesses. Early recognition and timely intervention are essential to prevent morbidity and improve clinical outcomes [66].

### 7.1. Endoscopic Management of Intestinal Strictures

Endoscopic techniques have emerged as valuable minimally invasive approaches for the management of intestinal strictures associated with tuberculosis. Endoscopic balloon dilation has been reported as a safe and effective method for treating short fibrotic strictures, particularly in patients who remain symptomatic despite adequate anti-tubercular therapy [66]. This procedure may relieve obstructive symptoms and reduce the need for surgical intervention. Endoscopic stricturotomy has also been explored as an alternative technique for treating fibrotic strictures. This method involves controlled incision of the stricture using endoscopic instruments and may provide symptomatic relief in selected patients [67,68]. Although most evidence regarding stricturotomy comes from studies in inflammatory bowel disease, similar principles have been applied in selected cases of intestinal tuberculosis-related strictures.

### 7.2. Surgical Indications

Surgical intervention is generally reserved for patients who develop severe complications or who fail to respond to medical therapy. Common indications for surgery include intestinal obstruction caused by fibrotic strictures, intestinal perforation, uncontrolled gastrointestinal bleeding, and intra-abdominal abscess formation [8]. In many cases, surgery is performed to address complications rather than to treat the underlying infection itself. Limited intestinal resection or stricturoplasty may be performed depending on the location and extent of disease involvement.

### 7.3. Laparoscopic and Minimally Invasive Surgery

Advances in minimally invasive surgery have expanded the role of laparoscopy in the management of abdominal tuberculosis. Laparoscopic procedures may be used for diagnostic purposes, such as obtaining peritoneal biopsies, or for therapeutic management of complications including adhesions, abscess drainage, or intestinal obstruction [69,70]. Compared with open surgery, laparoscopic approaches may offer several advantages, including reduced postoperative pain, shorter hospital stay, and faster recovery. However, surgical intervention should be carefully considered and reserved for patients with clear indications.

## 8. Biomarkers and Monitoring of Treatment Response

Monitoring treatment response in intestinal tuberculosis is essential for evaluating therapeutic effectiveness, detecting complications, and differentiating persistent disease from alternative diagnoses such as Crohn’s disease. In addition to clinical assessment and imaging, several laboratory biomarkers have been investigated to assist in disease monitoring and follow-up.

### 8.1. Fecal Biomarkers

Fecal biomarkers have emerged as useful non-invasive indicators of intestinal inflammation. Among these, fecal calprotectin is one of the most widely studied markers in gastrointestinal diseases. Calprotectin is a calcium-binding protein released from activated neutrophils and is considered a sensitive marker of intestinal mucosal inflammation [71]. Elevated fecal calprotectin levels have been reported in patients with intestinal tuberculosis, reflecting active intestinal inflammation. Studies suggest that fecal calprotectin may also help distinguish intestinal tuberculosis from pulmonary tuberculosis and other non-intestinal forms of the disease [72]. In addition, declining fecal calprotectin levels during therapy may indicate clinical improvement and mucosal healing. Serial measurement of fecal calprotectin during anti-tubercular therapy has also been proposed as a tool for monitoring treatment response. Reduction in biomarker levels during the early phase of treatment may correlate with favorable therapeutic outcomes and resolution of intestinal inflammation [73].

### 8.2. Serum Biomarkers

Serum biomarkers have also been evaluated for their diagnostic and monitoring utility in intestinal tuberculosis. Adenosine deaminase (ADA), an enzyme involved in purine metabolism and lymphocyte proliferation, has demonstrated particular value in the diagnosis of peritoneal tuberculosis. Elevated ADA levels in ascitic fluid, typically using a cutoff of 30–40 U/L, show high sensitivity (85–95%) and specificity (85–95%) for tuberculous peritonitis [33]. Serum ADA levels may also be elevated in intestinal tuberculosis, though with lower diagnostic accuracy compared to ascitic fluid measurements. Other serum inflammatory markers including C-reactive protein and erythrocyte sedimentation rate are commonly elevated in active ITB and may be useful for monitoring treatment response, with declining levels correlating with clinical improvement [48]. Interferon-gamma release assays such as QuantiFERON-TB Gold and T-SPOT.TB detect T-cell responses to M. tuberculosis-specific antigens and may support the diagnosis of latent or active tuberculosis infection, though they cannot reliably distinguish between pulmonary and extrapulmonary disease or between active and latent infection [74].

### 8.3. Emerging Biomarkers

In addition to calprotectin, other biomarkers have been explored for their potential role in the evaluation of intestinal inflammation and treatment monitoring. Lactoferrin and other fecal inflammatory markers have been studied as indicators of mucosal inflammation in gastrointestinal diseases, including inflammatory bowel disease and intestinal infections [71,75]. Although these biomarkers show promise as non-invasive monitoring tools, their role in intestinal tuberculosis remains under investigation. Additional studies are needed to determine their diagnostic accuracy and clinical utility in differentiating intestinal tuberculosis from other inflammatory bowel conditions.

### 8.4. Monitoring Treatment Response

Evaluation of treatment response typically involves a combination of clinical assessment, laboratory biomarkers, endoscopic findings, and radiologic imaging. Clinical improvement, including resolution of abdominal pain, fever, and obstructive symptoms, usually occurs within several weeks after initiation of anti-tubercular therapy. Endoscopic reassessment may demonstrate healing of mucosal ulcers and reduction in inflammatory lesions following treatment. Imaging modalities such as CT enterography or ultrasound may also be used to monitor regression of bowel wall thickening and lymphadenopathy. Overall, combining clinical evaluation with biomarker monitoring and imaging provides a comprehensive approach for assessing therapeutic response and identifying patients who may require further diagnostic evaluation or modification of treatment strategies.

## 9. Future Perspectives and Emerging Technologies

Advances in diagnostic technologies and precision medicine are expected to significantly improve the diagnosis and management of intestinal tuberculosis. Emerging approaches integrating molecular diagnostics, artificial intelligence, and advanced imaging techniques may enhance diagnostic accuracy and reduce delays in distinguishing intestinal tuberculosis from other gastrointestinal disorders, particularly Crohn’s disease.

### 9.1. Artificial Intelligence in Diagnostic Imaging

Artificial intelligence (AI) and machine learning techniques are increasingly being applied to medical imaging and endoscopic interpretation. Deep learning algorithms have demonstrated promising results in differentiating intestinal tuberculosis from Crohn’s disease by analyzing radiologic and endoscopic images. Multicenter studies using whole-slide histopathological image analysis have shown that AI-based systems can accurately identify morphological patterns that distinguish intestinal tuberculosis from inflammatory bowel disease [37]. Similarly, deep learning-based analysis of endoscopic images has been developed to assist clinicians in differentiating Crohn’s disease from intestinal tuberculosis. These systems analyze mucosal patterns and ulcer morphology to improve diagnostic accuracy and reduce interobserver variability among clinicians [38]. Machine learning models integrating clinical, radiologic, and endoscopic data have also demonstrated encouraging results in improving diagnostic precision in complex cases [39].

### 9.2. Innovations in Diagnostic Approaches for Intestinal Tuberculosis

Advances in digital health technologies and computational analysis are expected to further improve diagnostic capabilities in gastrointestinal diseases. Artificial intelligence-assisted imaging analysis may enhance pattern recognition, reduce interobserver variability among clinicians, and provide decision-support systems that facilitate early diagnosis of intestinal tuberculosis [76]. Future developments may also involve the integration of advanced molecular diagnostics, imaging analytics, and host-response biomarkers to establish more precise diagnostic algorithms. These approaches may contribute to earlier detection of intestinal tuberculosis and improve differentiation from inflammatory bowel diseases in complex clinical scenarios.

### 9.3. Precision Medicine Approaches

Precision medicine strategies aim to integrate clinical characteristics, imaging findings, molecular diagnostics, and host immune responses to guide individualized treatment decisions. Such approaches may improve diagnostic accuracy and help identify patients at higher risk of complications or treatment failure. Integration of advanced diagnostic techniques with personalized therapeutic strategies may also facilitate early stratification of patients and optimize clinical outcomes. As research in this field continues to evolve, the application of precision medicine in tuberculosis management may lead to improved diagnostic pathways and more targeted therapeutic interventions.

## 10. Conclusions

Intestinal tuberculosis remains a challenging form of extrapulmonary tuberculosis due to its nonspecific clinical presentation and significant overlap with other gastrointestinal disorders, particularly Crohn’s disease. Delayed or inaccurate diagnosis may lead to inappropriate treatment, disease progression, and the development of severe complications such as intestinal obstruction, perforation, and fistula formation. Accurate diagnosis requires a comprehensive and integrated approach that combines clinical evaluation with radiologic imaging, endoscopic examination, histopathological analysis, and microbiological or molecular diagnostic testing. Advances in diagnostic technologies, including polymerase chain reaction assays, GeneXpert testing, artificial intelligence-assisted imaging analysis, and next-generation sequencing, have improved diagnostic accuracy and may facilitate earlier identification of the disease. Medical management with standard anti-tubercular therapy remains the cornerstone of treatment and is effective in the majority of patients. However, a subset of individuals develops complications requiring endoscopic or surgical intervention. Minimally invasive techniques such as endoscopic balloon dilation and laparoscopic procedures may help reduce surgical morbidity when appropriately applied. Emerging diagnostic technologies, including artificial intelligence-based image analysis and advanced molecular platforms, may further enhance diagnostic precision and enable earlier differentiation between intestinal tuberculosis and other inflammatory bowel diseases. Continued research is needed to validate these technologies and to explore precision medicine approaches that integrate clinical, molecular, and imaging data. Overall, early recognition of intestinal tuberculosis, timely initiation of appropriate therapy, and multidisciplinary management are essential for improving patient outcomes and reducing disease-related complications.

## Figures and Tables

**Table 2:** Key Diagnostic Features Differentiating Intestinal Tuberculosis and Crohn’s Disease.

Feature	Intestinal Tuberculosis	Crohn’s Disease	References
Epidemiology	Common in TB-endemic regions	More common in Western countries	[2,11]
Systemic symptoms	Fever, night sweats, weight loss common	Less prominent systemic symptoms	[2,10]
Common location	Ileocecal region	Terminal ileum and colon	[1,2]
Disease distribution	Continuous involvement	Skip lesions common	[20,21,22]
Perianal disease	Rare	Common	[11,12]
Endoscopic ulcer pattern	Transverse ulcers	Longitudinal ulcers	[16,23]
Endoscopic appearance	Nodules, short strictures	Cobblestone mucosa	[23]
Stricture pattern	Short concentric strictures	Long fibrotic strictures	[8,9]
Lymph nodes (CT)	Necrotic lymphadenopathy	Non-necrotic nodes	[20,21,22]
Mesenteric fat	Usually absent	Creeping fat common	[20,21]
Histopathology	Caseating granulomas	Non-caseating granulomas	[40,44]
AFB staining/culture	May detect Mycobacterium tuberculosis	Negative	[6,7]
PCR/GeneXpert	May detect TB DNA	Negative	[6,27,41]
Response to therapy	Responds to anti-tubercular therapy	Requires immunosuppressive therapy	[16,35]

## Data Availability

No new data were created or analyzed in this study. Data sharing is not applicable to this article.

## Abbreviations

The following abbreviations are used in this manuscript:

Abbreviation	Full Term
ADA	Adenosine deaminase
AFB	Acid-fast bacilli
AI	Artificial intelligence
ATT	Anti-tubercular therapy
CFU	Colony-forming units
CRISPR	Clustered regularly interspaced short palindromic repeats
CT	Computed tomography
DNA	Deoxyribonucleic acid
EUS	Endoscopic ultrasound
GeneXpert	GeneXpert MTB/RIF (Xpert MTB/RIF rapid molecular assay)
HIV	Human immunodeficiency virus
IBD	Inflammatory bowel disease
ITB	Intestinal tuberculosis
LOD	Limit of detection
MDR-TB	Multidrug-resistant tuberculosis
MeSH	Medical Subject Headings
MTB	Mycobacterium tuberculosis
NGS	Next-generation sequencing
PCR	Polymerase chain reaction
PRISMA	Preferred Reporting Items for Systematic Reviews and Meta-Analyses
RIF	Rifampicin
TB	Tuberculosis
WHO	World Health Organization
XDR-TB	Extensively drug-resistant tuberculosis

## Appendix A

### Appendix A.1. Characteristics of Studies

**Study (Author, Year) [Ref]**	**Study Type**	**Key Findings Relevant to ITB**	**Limitations/Risk of Bias**
Zeng et al., 2023 [1]	Retrospective cohort	Ileocecal involvement in 82% of cases; CT enterography sensitivity 78%.	Single-center; retrospective design.
Choudhury et al., 2023 [2]	Comprehensive review	Necrotic nodes are key differentiator (OR 4.5) between ITB and Crohn’s.	Narrative review; potential selection bias.
Al-Zanbagi & Shariff, 2021 [3]	Systematic review	Pooled sensitivity of GeneXpert in ITB tissue was 64%.	Heterogeneity of included studies.
Maulahela et al., 2022 [5]	Review	Recent advances in ITB diagnosis including molecular and endoscopic tools.	Narrative summary; no quantitative synthesis.
Sasikumar et al., 2020 [6]	Diagnostic accuracy	GeneXpert sensitivity 64.3% in extrapulmonary TB.	Small sample for ITB subgroup.
Arora & Dhanashree, 2020 [7]	Diagnostic study	Smear sensitivity 12%, GeneXpert sensitivity 58% in paucibacillary samples.	Limited ITB-specific data.
Di Buono et al., 2024 [8]	Systematic review	Surgical resection required in 18% of complicated ITB cases; strictures most common indication.	Retrospective data pooling; heterogeneity.
Jena et al., 2023 [10]	Systematic review & meta-analysis	Strictures occur in 24% of ITB; 27% require endoscopic/surgical intervention.	High heterogeneity; variable definitions.
Kc et al., 2023 [11]	Case report & review	ITB mimics Crohn’s; misdiagnosis leads to inappropriate immunosuppression.	Single case; limited generalizability.
Sato et al., 2019 [12]	Case series	10 ITB cases initially misdiagnosed as IBD; ATT resolved all.	Small sample; retrospective.
Seo et al., 2017 [13]	Retrospective cohort	Misdiagnosis rate decreased from 54% to 18% after 2010 due to improved diagnostics.	Temporal confounding.
Nguyen et al., 2010 [14]	Review	Diagnosis of ITB relies on combination of clinical, imaging, and microbiological features.	Older data; narrative.
Al-Hadeedi et al., 1990 [15]	Retrospective review	Abdominal TB presentation and surgical management in endemic area.	Outdated diagnostic methods.
Pratap Mouli et al., 2017 [16]	Prospective cohort	Response to ATT at 2 months differentiates ITB from Crohn’s (PPV 94%).	Single-center; moderate sample.
Van Hoving et al., 2019 [18]	Cochrane systematic review	Abdominal ultrasound sensitivity 68%, specificity 72% for abdominal TB.	Limited ITB-specific data.
Aprile et al., 2024 [19]	Review	GI ultrasound useful for detecting bowel wall thickening and lymphadenopathy in ITB.	Emerging technique; limited validation.
Ma et al., 2019 [20]	Prospective cohort	CT enterography and ultrasound show mucosal healing in 73% at 6 months ATT.	Small sample (n = 89).
Sinan et al., 2002 [21]	Retrospective review	CT features of abdominal TB: necrotic nodes, ileocecal thickening, ascites.	Older CT technology.
Kalra et al., 2014 [22]	Retrospective study	MDCT enterography findings in small bowel TB: short strictures, mural thickening.	Retrospective.
Mehta et al., 2019 [23]	Review	Endoscopic and histologic features aiding ITB diagnosis.	Narrative; no new data.
Facciorusso et al., 2022 [24]	Systematic review & meta-analysis	EUS-FNB superior to FNA for lymph node tissue acquisition (OR 1.9).	Not ITB-specific.
Puri et al., 2012 [25]	Prospective study	EUS-FNA diagnostic yield 76% for tubercular lymphadenopathy.	Single-center; operator-dependent.
Pai et al., 2004 [26]	Systematic review	NAAT sensitivity 62% for tuberculous pleuritis.	Not ITB-specific.
Fei et al., 2021 [27]	Diagnostic study	Tissue TB antigen + Xpert sensitivity 88% for ITB vs. Crohn’s.	Single-center; moderate sample.
Reechaipichitkul et al., 2017 [28]	Comparative study	GeneXpert sensitivity 82% vs. smear 53% in pulmonary TB.	Pulmonary focus; limited ITB.
WHO, 2017 [29]	Technical report	Xpert Ultra LOD 15.6 CFU/mL vs. 112.6 for standard Xpert.	Not peer-reviewed primary study.
Dorman et al., 2018 [30]	Prospective multicenter diagnostic accuracy	Xpert Ultra sensitivity 63% in paucibacillary TB; LOD 15.6 CFU/mL.	Extrapulmonary TB limited; no ITB subgroup.
Peixoto et al., 2015 [31]	Review	Endoscopic biopsy techniques for GI diseases.	Not ITB-specific.
Shi et al., 2016 [32]	Review	Clinical and laboratory diagnosis of ITB.	Narrative; older literature.
Riquelme et al., 2006 [33]	Meta-analysis	Ascitic ADA sensitivity 93%, specificity 94% for tuberculous peritonitis.	Peritoneal TB focus; indirect ITB applicability.
Kreitmann et al., 2023 [34]	Review	Digital PCR and multiplex technologies for infectious disease diagnosis.	Not TB-specific; emerging technology.
Malekshoar et al., 2023 [35]	Methods study	CRISPR-Cas9 enrichment for mutation detection.	Not TB-specific.
Sibandze et al., 2022 [36]	Diagnostic study	Stool-based targeted sequencing sensitivity 83% for TB.	Pulmonary TB focus; limited ITB data.
Weng et al., 2022 [37]	Multicenter diagnostic study	AI whole-slide imaging distinguished ITB from Crohn’s with AUC 0.92.	Retrospective; external validation needed.
Kim et al., 2021 [38]	Diagnostic study	Deep learning endoscopic system AUC 0.95 for ITB vs. Crohn’s.	Single-country data; requires prospective validation.
Lu et al., 2021 [39]	Multicenter study	Machine learning integrated model AUC 0.96 for ITB vs. Crohn’s.	Retrospective; model complexity limits clinical translation.
Alvares et al., 2005 [40]	Retrospective cohort	Colonic TB endoscopic findings: ulcers (88%), nodules (49%).	Single-center; older data.
Jin et al., 2010 [41]	Retrospective comparative	TB-PCR sensitivity 68%, specificity 92% in ITB vs. Crohn’s.	Retrospective; small sample.
Makharia et al., 2010 [42]	Prospective comparative	Differentiating features: bleeding (OR 0.3 for ITB), transverse ulcers (OR 4.2).	Single-center.
Singh et al., 1996 [43]	Retrospective cohort	Colonic TB clinical and endoscopic profile.	Older data; limited diagnostics.
Pulimood et al., 1999 [44]	Prospective study	Caseating granulomas in 33% of ITB vs. 0% Crohn’s.	Single-center.
Amarapurkar et al., 2008 [45]	Prospective study	TB-PCR positive in 64% of ITB vs. 0% Crohn’s.	Small sample; single-center.
Sharma et al., 2017 [46]	Systematic review	Abdominal cocoon in TB; surgical management outcomes.	Rare condition; limited data.
Donovan et al., 2020 [47]	Randomized diagnostic accuracy	Xpert Ultra sensitivity 70% vs. Xpert 43% in TB meningitis.	Not ITB; indirect extrapolation.
Shen et al., 2013 [48]	Meta-analysis	Ascitic ADA sensitivity 93%, specificity 94% for tuberculous peritonitis.	Peritoneal TB focus.
Tao et al., 2014 [49]	Meta-analysis	Ascitic ADA diagnostic OR 56 for TB ascites.	Peritoneal TB focus.
Larsson et al., 2014 [50]	Cross-sectional	Fecal calprotectin median 210 mg/kg in ITB vs. 45 in pulmonary TB.	Small sample; single-center.
Limsrivilai et al., 2017 [51]	Meta-analysis	Bayesian model differentiating ITB from Crohn’s: sensitivity 83%, specificity 85%.	Heterogeneity of included studies.
Jellema et al., 2011 [52]	Systematic review	Diagnostic testing for IBD in primary care.	Not ITB-specific.
Aggarwal et al., 2017 [53]	Retrospective cohort	38% of ITB strictures showed poor response to ATT; required intervention.	Retrospective; selection bias.
Aljarallah, 2025 [54]	Clinical study	Response to short-course ATT in ITB.	Details limited (recent publication).
Park et al., 2009 [55]	Randomized trial	6-month ATT non-inferior to 9-month for ITB (response 95% vs. 97%).	Single-center; small sample.
Kempker et al., 2020 [56]	Cohort study	Bedaquiline/delamanid regimens effective in drug-resistant extrapulmonary TB.	Limited ITB-specific data.
WHO, 2020 [57]	Guideline	MDR-TB treatment recommendations: shorter all-oral regimens.	Guideline; not primary data.
Conradie et al., 2020 [58]	Clinical trial	BPaL regimen 90% success in XDR-TB.	Pulmonary TB focus.
Sharma et al., 2017 [59]	Randomized trial	Mycobacterium indicus pranii adjunct therapy improved cure rates in pulmonary TB.	Not ITB-specific.
Gupta et al., 2012 [60]	Experimental study	Immunotherapy with MIP as adjunct to TB chemotherapy.	Animal and in vitro; not ITB.
Mi et al., 2024 [61]	Review	Immunoregulatory compounds as adjuvant TB therapy.	Narrative; limited clinical data.
Soni et al., 2019 [62]	Systematic review & meta-analysis	Steroids reduced stricture risk in abdominal TB (RR 0.67).	Heterogeneity; limited ITB-specific data.
Kang et al., 2021 [63]	Case report	Intestinal perforation due to paradoxical reaction during ATT.	Single case.
Breen et al., 2004 [64]	Cohort study	Paradoxical reactions in 14% of HIV-negative TB patients.	Pulmonary TB predominant.
Sharma et al., 2021 [65]	Prospective observational	Serial fecal calprotectin discriminated ITB from Crohn’s during ATT trial.	Small sample; single-center.
Kumar et al., 2022 [66]	Case series	Endoscopic balloon dilation successful in 89% of tubercular strictures.	Small sample; retrospective.
Mohy-Ud-Din & Kochhar, 2020 [67]	Review	Endoscopic stricturotomy techniques for IBD strictures.	Not ITB-specific.
Jaber et al., 2024 [68]	Systematic review & meta-analysis	Endoscopic stricturotomy technical success 92%, clinical success 68%.	IBD focus; limited ITB data.
Wiggins et al., 2015 [69]	Systematic review	Laparoscopic adhesiolysis for small bowel obstruction.	Not TB-specific.
Bhandarkar & Bhanushali, 2003 [70]	Case report	Laparoscopic drainage of tuberculous abscess.	Single case.
Vernia et al., 2021 [71]	Review	Fecal calprotectin and lactoferrin as IBD biomarkers.	Not ITB-specific.
Larsson et al., 2014 [72]	Cross-sectional	Fecal calprotectin differentiated intestinal from pulmonary TB.	Small sample; single-center.
Jo et al., 2022 [73]	Prospective observational	Fecal calprotectin declined significantly after 2 months ATT (*p* < 0.001).	Small sample; single-center.
Pai et al., 2014 [74]	Systematic review	IGRA sensitivity 80%, specificity 79% for active TB.	Cannot distinguish active from latent TB.
Soubières & Poullis, 2016 [75]	Review	Emerging biomarkers for IBD diagnosis.	Not ITB-specific.
Sharma et al., 2020 [76]	Review	AI in diagnostic imaging: status and future opportunities.	Not ITB-specific.

### Appendix A.2. Categorized Keywords

**Category**	**Keywords**
Disease/Condition	Intestinal tuberculosis; abdominal tuberculosis; extrapulmonary tuberculosis; gastrointestinal tuberculosis
Diagnostic Modalities	Molecular diagnostics; endoscopy; colonoscopy; endoscopic ultrasound; computed tomography; CT enterography; abdominal ultrasound; histopathology; acid-fast bacilli staining; mycobacterial culture; polymerase chain reaction (PCR); GeneXpert MTB/RIF; GeneXpert MTB/RIF Ultra; adenosine deaminase (ADA); fecal calprotectin; interferon-gamma release assay (IGRA)
Emerging Technologies	Artificial intelligence; machine learning; deep learning; CRISPR; next-generation sequencing; multiplex PCR; digital health
Therapeutic Interventions	Anti-tubercular therapy; drug-resistant tuberculosis; multidrug-resistant tuberculosis (MDR-TB); extensively drug-resistant tuberculosis (XDR-TB); bedaquiline; delamanid; host-directed therapy; immunomodulation; paradoxical reaction
Surgical/Minimally Invasive	Minimally invasive surgery; laparoscopy; endoscopic balloon dilation; endoscopic stricturotomy; stricturoplasty; intestinal resection; surgical complications
Differential Diagnosis	Crohn’s disease; inflammatory bowel disease; granulomatous inflammation; caseating granuloma

## References

[1] Zeng J., Zhou G., Pan F. (2023). Clinical analysis of intestinal tuberculosis: A retrospective study. J. Clin. Med..

[2] Choudhury A., Dhillon J., Sekar A., Gupta P., Singh H., Sharma V. (2023). Differentiating gastrointestinal tuberculosis and Crohn’s disease: A comprehensive review. BMC Gastroenterol..

[3] Al-Zanbagi A.B., Shariff M.K. (2021). Gastrointestinal tuberculosis: A systematic review of epidemiology, presentation, diagnosis and treatment. Saudi J. Gastroenterol..

[4] Akgun Y. (2005). Intestinal and peritoneal tuberculosis: Changing trends over 10 years and a review of 80 patients. Can. J. Surg..

[5] Maulahela H., Simadibrata M., Nelwan E.J., Rahadiani N., Renesteen E., Suwarti S.W.T., Anggraini Y.W. (2022). Recent advances in the diagnosis of intestinal tuberculosis. BMC Gastroenterol..

[6] Sasikumar C., Utpat K., Desai U., Joshi J. (2020). Role of GeneXpert in the diagnosis of Mycobacterium tuberculosis. Adv. Respir. Med..

[7] Arora D., Dhanashree B. (2020). Utility of smear microscopy and GeneXpert for the detection of Mycobacterium tuberculosis in clinical samples. Germs.

[8] Di Buono G., Buscemi S., Lo Monte A.I., Geraci G., Romano G., Maienza E., Gulotta L., Amato G., Agrusa A. (2024). Surgical Management of Complicated Abdominal Tuberculosis: The First Systematic Review—New Treatments for an Ancient Disease and the State of the Art. J. Clin. Med..

[9] World Health Organization (2022). WHO Consolidated Guidelines on Tuberculosis: Module 4: Treatment, Drug-Resistant Tuberculosis Treatment, 2022 Update.

[10] Jena A., Mohindra R., Rana K., Neelam P.B., Thakur D.C., Singh H., Gupta P., Suri V., Sharma V. (2023). Frequency, outcomes, and need for intervention in stricturing gastrointestinal tuberculosis: A systematic review and meta-analysis. BMC Gastroenterol..

[11] Kc P., Bhattarai M., Adhikari S., Parajuli P., Bhandari S., Bhattarai H.B., Sharma N.K., Karki S., Acharya S., Basnet B. (2023). Intestinal Tuberculosis Can Masquerade as Crohn’s Disease: A Teachable Moment. SAGE Open Med. Case Rep..

[12] Sato R., Nagai H., Matsui H., Yamane A., Kawashima M., Higa K., Nakamura S., Ohshima N., Tamura A., Hebisawa A. (2019). Ten Cases of Intestinal Tuberculosis Which Were Initially Misdiagnosed as Inflammatory Bowel Disease. Intern. Med..

[13] Seo H., Lee S., So H., Kim D., Kim S.O., Soh J.S., Bae J.H., Lee S.H., Hwang S.W., Park S.H. (2017). Temporal Trends in the Misdiagnosis Rates Between Crohn’s Disease and Intestinal Tuberculosis. World J. Gastroenterol..

[14] Nguyen D.C., Do D.V., Do S.H., Nguyen V.X., Nguyen Q., Ninh V.K., Pham H.B., Huguier M. (2010). Diagnosis of Intestinal Tuberculosis at Viet Duc Hospital (2004–2009). Thai J. Surg..

[15] Al-Hadeedi S., Walia H.S., Al-Sayer H.M. (1990). Abdominal tuberculosis. Can. J. Surg..

[16] Pratap Mouli V., Munot K., Ananthakrishnan A., Kedia S., Addagalla S., Garg S.K., Benjamin J., Singla V., Dhingra R., Tiwari V. (2017). Endoscopic and clinical responses to anti-tubercular therapy differentiate intestinal tuberculosis from Crohn’s disease. Aliment. Pharmacol. Ther..

[17] Baumgart D.C. (2009). The diagnosis and treatment of Crohn’s disease and ulcerative colitis. Dtsch. Ärzteblatt Int..

[18] Van Hoving D.J., Griesel R., Meintjes G., Takwoingi Y., Maartens G., Ochodo E.A. (2019). Abdominal ultrasound for diagnosing abdominal tuberculosis or disseminated tuberculosis with abdominal involvement in HIV-positive individuals. Cochrane Database Syst. Rev..

[19] Aprile F., Vangeli M., Allocca M., Zilli A., Argollo M.C., D’Amico F., Parigi T.L., Danese S., Furfaro F. (2024). Gastrointestinal ultrasound in infectious diseases. Medicina.

[20] Ma L., Zhu Q., Li Y., Li W., Wang X., Liu W., Li J., Jiang Y. (2019). The potential role of CT enterography and gastrointestinal ultrasound in the evaluation of anti-tubercular therapy response of intestinal tuberculosis: A retrospective study. BMC Gastroenterol..

[21] Sinan T., Sheikh M., Ramadan S., Sahwney S., Behbehani A. (2002). CT features in abdominal tuberculosis: 20 years experience. BMC Med. Imaging.

[22] Kalra N., Agrawal P., Mittal V., Kochhar R., Gupta V., Nada R., Singh R., Khandelwal N. (2014). *Spectrum of* Imaging findings on MDCT enterography in small bowel tuberculosis. Clin. Radiol..

[23] Mehta V., Desai D., Abraham P., Rodrigues C. (2019). Making a positive diagnosis of intestinal tuberculosis with the aid of new biologic and histologic features: How far have we reached?. Inflamm. Intest. Dis..

[24] Facciorusso A., Crinò S.F., Gkolfakis P., Ramai D., Lisotti A., Papanikolaou I.S., Mangiavillano B., Tarantino I., Anderloni A., Fabbri C. (2022). Endoscopic ultrasound fine-needle biopsy versus fine-needle aspiration for lymph node tissue acquisition: A systematic review and meta-analysis. Gastroenterol. Rep..

[25] Puri R., Mangla R., Eloubeidi M., Vilmann P., Thandassery R., Sud R. (2012). Diagnostic yield of EUS-guided fine-needle aspiration and cytology in suspected tubercular intra-abdominal lymphadenopathy. Gastrointest. Endosc..

[26] Pai M., Flores L.L., Hubbard A., Riley L.W., Colford J.M. (2004). Nucleic acid amplification tests in the diagnosis of tuberculous pleuritis: A systematic review and meta-analysis. BMC Infect. Dis..

[27] Fei B., Zhou L., Zhang Y., Luo L., Chen Y. (2021). Application value of tissue tuberculosis antigen combined with Xpert MTB/RIF detection in differential diagnosis of intestinal tuberculosis and Crohn’s disease. BMC Infect. Dis..

[28] Reechaipichitkul W., Suleesathira T., Chaimanee P. (2017). Comparison of GeneXpert MTB/RIF assay with conventional AFB smear for diagnosis of pulmonary tuberculosis in northeastern Thailand. Southeast Asian J. Trop. Med. Public Health.

[29] World Health Organization (2017). WHO Meeting Report of a Technical Expert Consultation: Non-Inferiority Analysis of Xpert MTB/RIF Ultra Compared to Xpert MTB/RIF.

[30] Dorman S.E., Schumacher S.G., Alland D., Nabeta P., Armstrong D.T., King B., Hall S.L., Chakravorty S., Cirillo D.M., Tukvadze N. (2018). Xpert MTB/RIF Ultra for detection of Mycobacterium tuberculosis and rifampicin resistance: A prospective multicentre diagnostic accuracy study. Lancet Infect. Dis..

[31] Peixoto A., Silva M., Pereira P., Macedo G. (2015). Biopsies in gastrointestinal endoscopy: When and how. GE Port. J. Gastroenterol..

[32] Shi X.C., Zhang L.F., Zhang Y.Q., Liu X.Q., Fei G.J. (2016). Clinical and laboratory diagnosis of intestinal tuberculosis. Chin. Med. J..

[33] Riquelme A., Calvo M., Salech F., Valderrama S., Pattillo A., Arellano M., Arrese M., Soza A., Viviani P., Letelier L.M. (2006). Value of adenosine deaminase in ascitic fluid for the diagnosis of tuberculous peritonitis: A meta-analysis. J. Clin. Gastroenterol..

[34] Kreitmann L., Miglietta L., Xu K., Malpartida-Cardenas K., D’Souza G., Kaforou M., Brengel-Pesce K., Drick N., Eger K., Liu X. (2023). Next-generation molecular diagnostics: Leveraging digital technologies to enhance multiplexing in real-time PCR. TrAC Trends Anal. Chem..

[35] Malekshoar M., Azimi S.A., Kaki A., Mousazadeh L., Motaei J., Vatankhah M. (2023). CRISPR-Cas9 targeted enrichment and next-generation sequencing for mutation detection. J. Mol. Diagn..

[36] Sibandze D.B., Kay A., Dreyer V., Sikhondze W., Dlamini Q., DiNardo A., Mtetwa G., Lukhele B., Vambe D., Lange C. (2022). Rapid molecular diagnostics of tuberculosis resistance by targeted stool sequencing. Genome Med..

[37] Weng F., Meng Y., Lu F., Wang Y., Wang W., Xu L., Cheng D., Zhu J. (2022). Differentiation of intestinal tuberculosis and Crohn’s disease through an explainable machine learning method. Sci. Rep..

[38] Kim J.M., Kang J.G., Kim S., Cheon J.H. (2021). Deep-Learning System for Real-Time Differentiation Between Crohn’s Disease, Intestinal Behçet’s Disease, and Intestinal Tuberculosis. J. Gastroenterol. Hepatol..

[39] Lu Y., Chen Y., Peng X., Yao J., Zhong W., Li C., Zhi M. (2021). Development and Validation of a New Algorithm Model for Differential Diagnosis Between Crohn’s Disease and Intestinal Tuberculosis: A Combination of Laboratory, Imaging and Endoscopic Characteristics. BMC Gastroenterol..

[40] Alvares J.F., Devarbhavi H., Makhija P., Rao S., Kottoor R. (2005). Clinical, colonoscopic, and histological profile of colonic tuberculosis in a tertiary hospital. Endoscopy.

[41] Jin X.J., Kim J.M., Kim H.K., Kim L., Choi S.J., Park I.S., Han J.Y., Chu Y.C., Song J.Y., Kwon K.S. (2010). Histopathology and TB-PCR kit analysis in differentiating the diagnosis of intestinal tuberculosis and Crohn’s disease. World J. Gastroenterol..

[42] Makharia G.K., Srivastava S., Das P., Goswami P., Singh U., Tripathi M., Deo V., Aggarwal A., Tiwari R.P., Sreenivas V. (2010). Clinical, endoscopic, and histological differentiations between Crohn’s disease and intestinal tuberculosis. Am. J. Gastroenterol..

[43] Singh V., Kumar P., Kamal J., Prakash V., Vaiphei K., Singh K. (1996). Clinicocolonoscopic profile of colonic tuberculosis. Am. J. Gastroenterol..

[44] Pulimood A.B., Ramakrishna B.S., Kurian G., Peter S., Patra S., Mathan V.I., Mathan M.M. (1999). Endoscopic mucosal biopsies are useful in distinguishing granulomatous colitis due to Crohn’s disease from tuberculosis. Gut.

[45] Amarapurkar D.N., Patel N.D., Rane P.S. (2008). Diagnosis of Crohn’s disease in India where tuberculosis is widely prevalent. World J. Gastroenterol..

[46] Sharma V., Singh H., Mandavdhare H.S. (2017). Tubercular abdominal cocoon: Systematic review of an uncommon form of tuberculosis. Surg. Infect..

[47] Donovan J., Thu D.D.A., Phu N.H., Dung V.T.M., Quang T.P., Nghia H.D.T., Oanh P.K.N., Nhu T.B., Chau N.V.V., Ny N.T.H. (2020). Xpert MTB/RIF Ultra versus Xpert MTB/RIF for the diagnosis of tuberculous meningitis: A prospective, randomised, diagnostic accuracy study. Lancet Infect. Dis..

[48] Shen Y.C., Wang T., Chen L., Yang T., Wan C., Hu Q.J., Wen F.Q. (2013). Diagnostic accuracy of adenosine deaminase for tuberculous peritonitis: A meta-analysis. Arch. Med. Sci..

[49] Tao L., Ning H.J., Nie H.M., Guo X.Y., Qin S.Y., Jiang H.X. (2014). Diagnostic value of adenosine deaminase in ascites for tuberculosis ascites: A meta-analysis. Diagn. Microbiol. Infect. Dis..

[50] Larsson G., Shenoy K.T., Ramasubramanian R., Thayumanavan L., Balakumaran L.K., Bjune G.A., Moum B.A. (2014). Routine diagnosis of intestinal tuberculosis and Crohn’s disease in Southern India. World J. Gastroenterol..

[51] Limsrivilai J., Shreiner A.B., Pongpaibul A., Laohapand C., Boonanuwat R., Pausawasdi N., Pongprasobchai S., Manatsathit S., Higgins P.D.R. (2017). Meta-analytic Bayesian model for differentiating intestinal tuberculosis from Crohn’s disease. Am. J. Gastroenterol..

[52] Jellema P., van Tulder M.W., van der Horst H.E., Florie J., Mulder C.J., van der Windt D.A. (2011). Inflammatory bowel disease: A systematic review on the value of diagnostic testing in primary care. Color. Dis..

[53] Aggarwal P., Kedia S., Sharma R., Bopanna S., Madhusudhan K.S., Yadav D.P., Goyal S., Mouli V.P., Sahni P., Das P. (2017). Tubercular intestinal strictures show poor response to anti-tuberculous therapy. Dig. Dis. Sci..

[54] Aljarallah B.M. (2025). Clinical study of intestinal tuberculosis and its response to short-course anti-tuberculosis therapy. J. Pioneer. Med. Sci..

[55] Park S.H., Yang S.K., Yang D.H., Kim K.J., Yoon S.M., Choe J.W., Ye B.D., Byeon J.S., Myung S.J., Yoon Y.S. (2009). Prospective randomized trial of six-month versus nine-month therapy for intestinal tuberculosis. Antimicrob. Agents Chemother..

[56] Kempker R.R., Mikiashvili L., Zhao Y., Benkeser D., Barbakadze K., Bablishvili N., Avaliani Z., Peloquin C.A., Blumberg H.M., Kipiani M. (2020). Clinical Outcomes Among Patients with Drug-resistant Tuberculosis Receiving Bedaquiline- or Delamanid-Containing Regimens. Clin. Infect. Dis..

[57] World Health Organization (2020). WHO Consolidated Guidelines on Tuberculosis: Module 4: Treatment, Drug-Resistant Tuberculosis Treatment.

[58] Conradie F., Diacon A.H., Ngubane N., Howell P., Everitt D., Crook A.M., Mendel C.M., Egizi E., Moreira J., Timm J. (2020). Treatment of highly drug-resistant pulmonary tuberculosis. N. Engl. J. Med..

[59] Sharma S.K., Katoch K., Sarin R., Balambal R., Kumar Jain N., Patel N., Murthy K.J.R., Singla N., Saha P.K., Khanna A. (2017). Efficacy and safety of Mycobacterium indicus pranii as an adjunct therapy in Category II pulmonary tuberculosis: A randomized trial. Sci. Rep..

[60] Gupta A., Ahmad F.J., Ahmad F., Gupta U.D., Natarajan M., Katoch V., Bhaskar S. (2012). Immunotherapy with Mycobacterium indicus pranii as an adjunct to chemotherapy for tuberculosis. PLoS ONE.

[61] Mi J., Wu X., Liang J. (2024). Advances in adjuvant therapy for tuberculosis with immunoregulatory compounds. Front. Microbiol..

[62] Soni H., Bellam B.L., Rao R.K., Kumar P.M., Mandavdhare H.S., Singh H., Sharma V. (2019). Use of steroids for abdominal tuberculosis: A systematic review and meta-analysis. Infection.

[63] Kang S.H., Moon H.S., Park J.H., Kim J.S., Kang S.H., Lee E.S., Kim S.H., Lee B.S., Sung J.K., Jeong H.Y. (2021). Intestinal Perforation as a Paradoxical Reaction to Antitubercular Therapy: A Case Report. Ann. Coloproctology.

[64] Breen R.A., Smith C.J., Bettinson H., Dart S., Bannister B., Johnson M.A., Lipman M.C. (2004). Paradoxical reactions during tuberculosis treatment in patients with and without HIV coinfection. Thorax.

[65] Sharma V., Verma S., Kumar-M P., Mandavdhare H.S., Singh H., Shah J., Kalsi D., Dutta A., Mishra S., Prasad K.K. (2021). Serial measurements of faecal calprotectin may discriminate intestinal tuberculosis and Crohn’s disease in patients started on antitubercular therapy. Eur. J. Gastroenterol. Hepatol..

[66] Kumar P., Jena A., Birda C.L., Mandavdhare H.S., Singh H., Gupta P., Prasad K.K., Sharma V. (2022). Safety and efficacy of non-fluoroscopic endoscopic dilatation of gastrointestinal tuberculosis related strictures. BMC Gastroenterol..

[67] Mohy-Ud-Din N., Kochhar G.S. (2020). Endoscopic stricturotomy for management of strictures in inflammatory bowel disease. Crohns Colitis 360.

[68] Jaber F., Numan L., Ayyad M., Almeqdadi M., Altayar O., Christein J., Hashash J.G., Farraye F.A. (2024). Efficacy and safety of endoscopic stricturotomy in inflammatory bowel disease-related strictures: A systematic review and meta-analysis. Dig. Dis. Sci..

[69] Wiggins T., Markar S.R., Harris A. (2015). Laparoscopic adhesiolysis for acute small bowel obstruction: Systematic review and pooled analysis. Surg. Endosc..

[70] Bhandarkar D., Bhanushali P. (2003). Laparoscopic drainage of a peripancreatic tuberculous abscess. Surg. Endosc..

[71] Vernia F., Viscido A., Di Ruscio M., Stefanelli G., Valvano M., Latella G. (2021). Fecal Lactoferrin and Other Putative Fecal Biomarkers in Crohn’s Disease: Do They Still Have a Potential Clinical Role?. Digestion.

[72] Larsson G., Shenoy K.T., Ramasubramanian R., Balakumaran L.K., Bjune G.A., Moum B.A. (2014). Faecal calprotectin levels differentiate intestinal from pulmonary tuberculosis: An observational study from Southern India. United Eur. Gastroenterol. J..

[73] Jo H.H., Kim E.Y., Jung J.T., Kwon J.G., Kim E.S., Lee H.S., Lee Y.J., Kim K.O., Jang B.I. (2022). Value of fecal calprotectin measurement during the initial period of therapeutic anti-tubercular trial. Clin. Endosc..

[74] Pai M., Denkinger C.M., Kik S.V., Rangaka M.X., Zwerling A., Oxlade O., Metcalfe J.Z., Cattamanchi A., Dowdy D.W., Dheda K. (2014). Gamma interferon release assays for detection of Mycobacterium tuberculosis infection. Clin. Microbiol. Rev..

[75] Soubières A.A., Poullis A. (2016). Emerging role of novel biomarkers in the diagnosis of inflammatory bowel disease. World J. Gastrointest. Pharmacol. Ther..

[76] Sharma P., Suehling M., Flohr T., Comaniciu D. (2020). Artificial intelligence in diagnostic imaging: Status quo, challenges, and future opportunities. J. Thorac. Imaging.

